# Optimal Designs of SVC-Based Content Placement and Delivery in Wireless Caching Networks

**DOI:** 10.3390/s23104823

**Published:** 2023-05-17

**Authors:** Xuewei Zhang, Lin Zhang, Yuan Ren, Jing Jiang, Junxuan Wang

**Affiliations:** School of Communications and Information Engineering, Xi’an University of Posts and Telecommunications, Xi’an 710121, China

**Keywords:** wireless caching, content placement, content transmission, scalable video coding, beamforming

## Abstract

To allieviate the heavy traffic burden over backhaul links and improve the user’s quality of service (QoS), edge caching plays an important role in wireless networks. This paper investigated the optimal designs of content placement and transmission in wireless caching networks. The contents to be cached and requested were encoded into individual layers by scalable video coding (SVC), and different sets of layers can provide different viewing qualities to end users. The demanded contents were provided by helpers caching the requested layers, or by the macro-cell base station (MBS) otherwise. In the content placement phase, this work formulated and solved the delay minimization problem. In the content transmission phase, the sum rate optimization problem was established. To effectively solve the nonconvex problem, the methods of semi-definite relaxation (SDR), successive convex approximation (SCA), and arithmetic-geometric mean (AGM) inequality were adopted, after which the original problem was transformed into the convex form. The numerical results show that the transmission delay is reduced by caching contents at helpers. Moreover, the fast convergence of the proposed algorithm for solving the sum rate maximization problem is presented, and the sum rate gain of edge caching is also revealed, as compared to the benchmark scheme without content caching.

## 1. Introduction

Recently, the explosion of mobile Internet and diversified multimedia services has led to a higher quality of service (QoS) requirements in terms of system throughput, transmission delay, and massive connectivity [1]. To address these challenges, wireless caching is considered as a promising enabling technique, and is gaining more and more attention in the development of the sixth generation (6G) networks [2], such as in the space–air–ground integrated networks (SAGIN). This architecture interconnects satellites, aerial platforms, and ground communication systems, which can meet the strict requirements of wide coverage and flexible connectivity [3]. In addition, the SAGIN is capable of resisting natural disasters and providing high bandwidth. This is beneficial for providing reliable video services, especially in some remote areas. It has some advantages that traditional ground networks cannot provide.

Video traffic typically has a strong characteristic of redundancy, so we can improve the efficiency of traffic transmission by wireless caching [4]. With the aid of wireless caching, the popular contents can be delivered to users closer and faster. The cache placement phase is executed during off-peak periods, and the content transmission is performed when content requests occur. Moreover, edge caching enables additional performance gains [5]. The transmission distance for delivering contents from a network becomes shorter than that from the remote centralized server. In [6], the authors investigated proactive caching in cloud radio access networks (CRANs). In [7], the authors proposed a novel secure random caching scheme for large-scale multi-antenna heterogeneous wireless networks, and demonstrated that edge caching could improve the user satisfaction and the achievable network throughput. In [8], the authors proposed to improve the quality of experience (QoE) for users when offering multimedia videos by using software-defined intra network caching and computing in the SAGIN. In [9], the authors explored a new SAGIN architecture to support these new requirements of 6G mobile communication networks in a flexible, low-latency, and efficient manner.

Due to the backhaul capacity limits and the changing user needs, there is an increasing demand for multi-quality video services. For example, some people need standard definition videos (SDVs) with less latency when watching news sports, while needing high definition videos (HDVs) when watching movies. Scalable video coding (SVC) is then designed. Each video file is divided into a base layer (BL) and several enhancement layers (ELs). The layered design of SVC can meet the needs of different video qualities. The video quality can be gradually enhanced by adding more layers. BL can be used to decode the video with the lowest viewing quality, i.e., SDV, and the continuous BL and ELs can provide HDV. Meanwhile, SVC is able to avoid delivering unnecessary layers, which reduces the transmission latency and backhaul burden. With different channel conditions, the users can also determine the exact number of received SVC layers.

**Figure 1:**
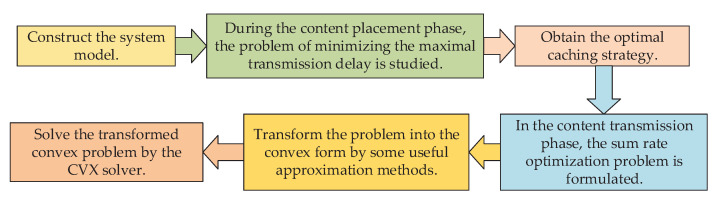
The overall framework of this paper.

This research investigated the optimal designs of content placement and transmission in caching-enabled networks. The helpers are capable of caching and transmitting SVC-based layers, and end users will turn to the macro-cell base station (MBS) for content transmission when requested contents are not available locally at helpers. The overall framework of this paper is shown in Figure 1. The main contributions of this paper are summarized as follows.

• Considering the different time-scales of content caching and transmission, this paper proposed a two-stage design. During the content placement phase, this work studied the optimal caching strategy to minimize the maximal transmission delay. Then, for the transmission phase, we formulated and solved the sum rate optimization problem, and then transformed the problem into the convex form by some useful approximation methods.
• To provide multi-level viewing qualities to end users, SVC was introduced into the content caching and transmission designs. The SVC-based layers are cached in the local caches of helpers, and the layer sets with the different numbers of layers can be provided to users depending on their preferences.
• In the content transmission phase, the sum rate optimization problem was formulated, which is non-convex. To effectively solve the problem, some useful transformation methods were adopted, i.e., semi-definite relaxation (SDR), successive convex approximation (SCA), and arithmetic-geometric mean (AGM) inequality. The nonconvex problem was then transformed into a convex problem and solved by the CVX solver. Additionally, this paper also performed detailed performance evaluations to demonstrate the effectiveness of the proposed algorithm.

The remainder of this paper is organized as follows. Section 3 provides the system model. The content placement and transmission designs are presented in Section 4 and Section 5, respectively. Simulation results are given in Section 6. Finally, conclusions and future work are presented in Section 7.

## 2. Related Work

With the help of wireless caching, some edge devices with finite caching capacities between users and content server are deployed. Content placement focuses on delivering files to edge devices during off-peak time periods to yield lower delivery latency. During the idle period of the network, this work performs content placement to obtain the optimal caching strategy. The optimization problem of content placement for cooperative caching was studied in [10]. In [11], the authors investigated the popularity-based content caching at network edges. The influence of unknown user density and content popularity on the cache placement at edge servers was studied in [12]. In [13], the authors proposed an optimized content placement method for enabling drone networks based on the spatiotemporal distribution characteristics of content popularity, remaining cache changes, and user download experience effects.

Apart from the content placement schemes, the content transmission designs have also been studied in the literature. In [14], the authors studied the design of joint content push and transmission based on caching helpers. The sum rate maximization problem was formulated and solved. In [15], the authors investigated a new wireless caching method and proposed two non-orthogonal multiple access (NOMA) enabled caching strategies. However, these works failed to consider the joint design of cache placement and content delivery.

Combining SVC with edge caching allows users to request video files of different sharpnesses. In [16], the authors investigated SVC based video retrieval in cache-aided device-to-device (D2D) networks. In [17], a NOMA-enhanced SVC multicast scheme for cellular networks was studied. In addition, Guo et al. [18] designed the mulitcast beamforming for SVC-based content transmission. The authors designed two SVC-aware probability caching strategies, which use cached data blocks to determine the path from the client to the server based on the SVC encoded video blocks and the location of the content router in [19].

The aforementioned studies failed to consider the joint design of content cache and transmission when providing multiple viewing qualities. To fill this void, this paper investigated the optimal designs of content placement and transmission for SVC-based files.

## 3. System Model

This section introduces the system model of the considered scenario, including the wireless caching protocol and the content transmission protocol.

### 3.1. Wireless Caching Protocol

**Figure 2:**
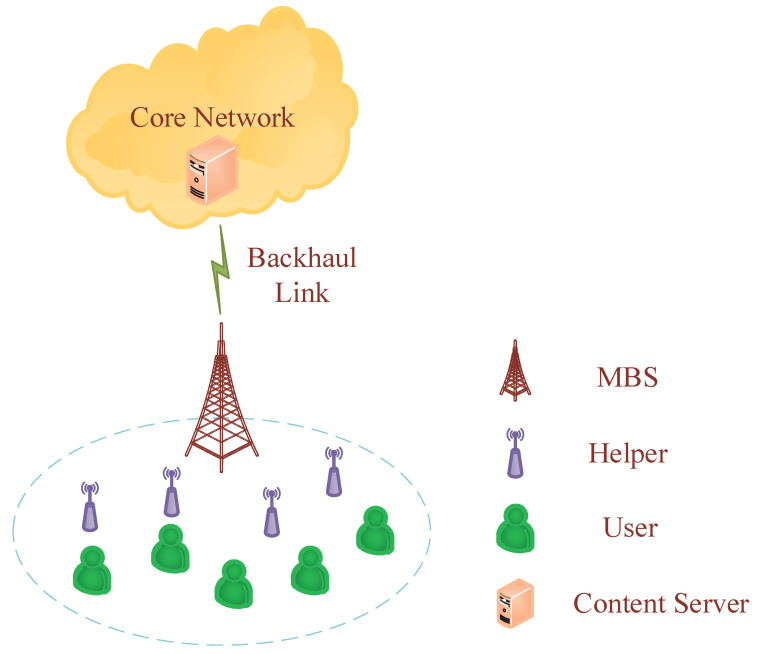
In the considered network, we investigated the downlink scenario for content transmissions, including *M* helpers and *K* users. Assume all helpers cache the same files.

This paper considered a network scenario of one MBS and *M* edge nodes, also called helpers in this paper. The edge nodes were equipped with caching capabilities, as shown in Figure 2. Due to the limitation of cache capacity, each helper cached some files according to the designed caching scheme. The MBS served *K* single-antenna users. Each caching helper was equipped with *T* antennas, and the MBS was equipped with $T_{B}$ antennas. There were *F* files, and for the sake of analysis, it was assumed that all files had the same size $S_{f}$ in Mbits. Each file was encoded into *L* layers, and the size of the *l*-th layer from the *f*-th video was $s_{f,l}$, i.e., $S_{f}=\sum_{l=1}^{L}s_{f,l}$. The files were arranged in the descending order of request popularities. To meet the multi-quality viewing qualities of multimedia videos, the Mandelbrot–Zipf (M-Zipf) law was developed for cellular video requests [20], where the request probability of the *f*-th video is

(1) $$\begin{array}{c} p\left(f\right)=\frac{{\left(f+q\right)}^{-\alpha }}{\sum_{n=1}^{F}{\left(n+q\right)}^{-\alpha }},f=1,2,\ldots ,F, \end{array}$$

where α is the skewness parameter to account for the degree of request concentration [21] and *q* is the plateau factor. The higher the value of *q*, the smaller the difference among the request probabilities of the most popular files. If q=0, the M-Zipf distribution becomes the widely used Zipf distribution.

The request preference of SDV for the *f*-th video is $p_{SDV}\left(f\right)=\frac{f-1}{F-1}$ [22], and therefore the preference for HDV is $p_{HDV}\left(f\right)=1-p_{SDV}\left(f\right)$. When HDV is requested, it is supposed that all ELs share the same popularity. To this end, the request probability of the *l*-th layer of the *f*-th video is expressed as

(2) $$\begin{array}{c} p_{f,l}=\left\{\begin{array}{c} p\left(f\right)\cdot \frac{f-1}{F-1},\;\;\;\;\;\;\;\;\;\;l=1;\\ p\left(f\right)\cdot \frac{F-f}{\left(F-1)(L-1\right)},l=2,\ldots ,L. \end{array}\right. \end{array}$$

It is supposed that each user has the information about the channel conditions and can request the appropriate number of layers based on the channel conditions. This research considered the case where helpers work independently from each other and focus on the multi-user scenario. Each helper caches parts of the SVC-based layers according to the designed cache placement scheme, and each user requests at most one file at a time. This work also assumed that each user requests a different file at the same moment.

When the user generates a file request, the request will be intercepted by helpers, and the helpers then check whether the requested one is cached in its storage. If the content is already cached, the requested one can be readily delivered from the helpers. Otherwise, the MBS covering the users will satisfy the user requests.

### 3.2. Content Transmission Protocol

When the user *k* requests layer $l_{k}$ from the helper, the helpers send the user the signal $x_{k,l_{k}}=w_{k,l_{k}}x_{k,l_{k}}$, where $w_{k,l_{k}}\overset{\Delta }{\;=}{\left[w_{k,l_{k}}^{1},w_{k,l_{k}}^{2},\ldots ,w_{k,l_{k}}^{M}\right]}^{H}\in {}^{MT\times 1}$ is the aggregated beamforming vector from all helpers; $w_{k,l_{k}}^{i}$ is the beamforming vector from the *i*-th helper for user *k* when delivering layer $l_{k}$; $x_{k,l_{k}}$ is the desired data symbol for the user, satisfying $E[|x_{k,l_{k}}{|}^{2}]=1$. The received signal at user *k* for delivering layer $l_{k}$ is denoted as

(3) $$\begin{array}{c} y_{k,l_{k}}=h_{k}x_{k,l_{k}}+\sum_{k^{\prime }=1,k^{\prime }\neq k}^{K}\sum_{l_{k^{\prime }}=1}^{L_{k^{\prime }}}h_{k}x_{k^{\prime },l_{k^{\prime }}}+\sum_{l=1,l\neq l_{k}}^{L_{k}}h_{k}x_{k,l}+n_{k}, \end{array}$$

where $h_{k}≜\left[{\left(h_{k}^{1}\right)}^{H},{\left(h_{k}^{2}\right)}^{H},\ldots ,{\left(h_{k}^{M}\right)}^{H}\right]\in C^{1\times MT}$ is the channel gain between helpers and user *k*, and $h_{k}^{i}$ denotes the channel gain from the *i*-th helper; $z_{k}$(k=1,2,…,K) denotes additive Gaussian white noise (AWGN); and $L_{k}$ indicates the number of video layers requested by user *k*. The received signal-to-interference-plus-noise ratio (SINR) is

$$\mathrm{SIN}R_{k,l_{k}}=\frac{|h_{k}w_{k,l_{k}}{|}^{2}}{\sum_{k^{\prime }=1,k^{\prime }\neq k}^{K}\sum_{l_{k^{\prime }}=1}^{L_{k^{\prime }}}|h_{k}w_{k^{\prime }l_{k^{\prime }}}{|}^{2}+\sum_{l=1,l\neq l_{k}}^{L_{k}}|h_{k}w_{k,l}{|}^{2}+\sigma_{k}^{2}},$$

where $\sigma_{k}^{2}$ is the AWGN variance of user *k*.

When the file requested by the user is not cached at the helpers, the content will be provided by the MBS covering the user. After the user sends a request, the MBS sends the signal $x_{{}_{k,l_{k}}}^{B}=w_{k,l_{k}}^{B}x_{k,l_{k}}^{B}$ to the user, where $w_{{}_{k,l_{k}}}^{B}\in C^{T_{B}\times 1}$ is the aggregated beamforming vector from the MBS. $x_{k,l_{k}}^{B}$ is the desired data symbol for user *k* when layer *l* is demanded, satisfying $E[|x_{k,l_{k}}^{B}{|}^{2}]=1$. The received signal at user *k* is denoted as

(4) $$\begin{array}{c} y_{{}_{k,l_{k}}}^{B}=h_{k}^{B}x_{{}_{k,l_{k}}}^{B}+\sum_{k^{\prime }=1,k^{\prime }\neq k}^{K}\sum_{l_{k^{\prime }}=1}^{L_{k^{\prime }}}h_{k}^{B}x_{k^{\prime },l_{k^{\prime }}}^{B}+\sum_{l=1,l\neq l_{k}}^{L_{k}}h_{k}^{B}x_{k,l}^{B}+z_{{}_{k}}^{B}, \end{array}$$

where $h_{{}_{k}}^{B}\in C^{1\times T_{B}}$ is the channel gain between the MBS and user *k* and $z_{{}_{k}}^{B}$ denotes the AWGN. The received SINR from the serving MBS is therefore given by

$${\mathrm{SINR}}_{k,l_{k}}^{B}=\frac{|h_{k}^{B}w_{{}_{k,l_{k}}}^{B}{|}^{2}}{\sum_{k^{\prime }=1,k^{\prime }\neq k}^{K}\sum_{l_{k^{\prime }}=1}^{L_{k^{\prime }}}|h_{k}^{B}w_{{}_{k^{\prime },l_{k^{\prime }}}}^{B}{|}^{2}+\sum_{l=1,l\neq l_{k}}^{L_{k}}|h_{k}^{B}w_{{}_{k,l}}^{B}{|}^{2}+{\left(\sigma_{k}^{B}\right)}^{2}},$$

where ${\left(\sigma_{k}^{B}\right)}^{2}$ is the AWGN variance of user *k*.

## 4. Optimal Design for Content Placement

In this section, this paper intended to propose the content placement scheme to minimize the maximal transmission delay resulted from retrieving each layer. We wrote the worst-case delay $D=\max_{f,l}p_{f,l}\left[z_{f,l}\frac{s_{f,l}}{R_{h}}+\left(1-z_{f,l}\right)\left(\frac{s_{f,l}}{R_{b}}+\frac{s_{f,l}}{R_{m}}\right)\right]$, and then minimized the maximum delay. Based on the above assumptions, the content placement problem for SVC-based layer caching was formulated as

(5a) $$\begin{array}{cc} \; & \min_{z_{f,l}}\;\;D \end{array}$$

(5b) $$\begin{array}{cc} \; & s.t.\;\;\sum_{f=1}^{F}\sum_{l=1}^{L}z_{f,l}s_{f,l}\leq C_{h}, \end{array}$$

(5c) $$\begin{array}{cc} \; & \;\;\;\;\;\;\;z_{f,l}\in \left\{0,1\right\}, \end{array}$$

where $R_{h}$, $R_{m}$, and $R_{b}$ represent the transmission rates of helpers, the MBS, and the backhaul link, respectively; $C_{h}$ indicates the cache size of the helper; and $z_{f,l}$ represents the binary caching decision of the *l*-th layer from the *f*-th video. (5b) is the cache capacity constraint of each helper, and (5c) gives the feasible range of the caching variable.

Then, slack variable *t*, satisfying D≤t, was introduced, and the optimization problem became

(6a) $$\begin{array}{cc} \; & \min\;t \end{array}$$

(6b) $$\begin{array}{cc} \; & s.t.\;\;\sum_{f=1}^{F}\sum_{l=1}^{L}z_{f,l}s_{f,l}\leq C_{h}, \end{array}$$

(6c) $$\begin{array}{cc} \; & \;\;\;\;\;\;\;z_{f,l}\in \left\{0,1\right\}, \end{array}$$

(6d) $$\begin{array}{cc} \; & \;\;\;\;\;\;\;p_{f,l}\left[z_{f,l}\frac{s_{f,l}}{R_{h}}+\left(1-z_{f,l}\right)\left(\frac{s_{f,l}}{R_{b}}+\frac{s_{f,l}}{R_{m}}\right)\right]\leq t. \end{array}$$

The transformed problem (6) is convex, and we can easily solve the problem using the CVX solver. Then, the optimal value $z_{f,l}^{*}$ was obtained.

## 5. Optimal Design for Content Delivery

### 5.1. Problem Formulation

During the content transmission phase, this work planned to maximize the sum rate while meeting the users’ minimum QoS requirements [14]. After solving problem (6), the optimal solution for $z_{f,l}$ was obtained. The sum rate can then be denoted as

(7) $$\begin{array}{cc} R & =\sum_{k=1}^{K}\sum_{l_{k}=1}^{L_{k}}[z_{f(k),l_{k}}^{*}\log_{2}\left(1+{\mathrm{SINR}}_{k,l_{k}}\right)+\left(1-z_{f(k),l_{k}}^{*}\right)\log_{2}\left(1+{\mathrm{SINR}}_{k,l_{k}}^{B}\right)], \end{array}$$

where f(k) denotes the file requested by user *k*.

To maximize (7), the optimal beamforming vectors should be found by solving the following optimization problem:

(8a) $$\begin{array}{cc} \; & \max_{\left\{w_{k,l_{k}},w_{k,l_{k}}^{B}\right\}}R \end{array}$$

(8b) $$\begin{array}{cc} \; & s.t.\;{\mathrm{SINR}}_{k,l_{k}}\geq \mu_{k,l_{k}}, \end{array}$$

(8c) $$\begin{array}{cc} \; & \;\;\;\;\;\;{\mathrm{SINR}}_{k,l_{k}}^{B}\geq \mu_{k,l_{k}}^{B}, \end{array}$$

(8d) $$\begin{array}{cc} \; & \;\;\;\;\;\sum_{k=1}^{K}\sum_{l_{k}=1}^{L_{k}}|w_{{}_{k,l_{k}}}^{i}{|}^{2}\leq P_{\max},\;\forall i=1,2,\ldots ,M, \end{array}$$

(8e) $$\begin{array}{cc} \; & \;\;\;\;\;\sum_{k=1}^{K}\sum_{l_{k}=1}^{L_{k}}|w_{{}_{k,l_{k}}}^{B}{|}^{2}\leq P_{\max}^{B}. \end{array}$$

In problem (8), when providing layer $l_{k}$ to the *k*-th user, we designed the optimal beamforming vectors from the helpers and MBS, denoted as $w_{k,l_{k}}$ and $w_{k,l_{k}}^{B}$, by maximizing the sum rate of users. Constraints (8b) and (8c) guarantee the minimum QoS requirements, where $\mu_{k,l_{k}}$ and $\mu_{k,l_{k}}^{B}$ are the pre-defined QoS thresholds for helpers and the MBS if user *k* requests layer $l_{k}$. Constraint (8d) is the maximum power constraint of helpers, and (8e) is the power constraint of the MBS, where $P_{\max}$ and $P_{\max}^{B}$ denote the maximum transmit powers of the helper and the MBS, respectively, satisfying $P_{\max}<P_{\max}^{B}$.

### 5.2. Proposed Algorithm for Sum Rate Maximization

It is easily observed that the proposed optimization problem is non-convex. In order to transform the problem into a convex one, this paper firstly turned to the SDR method. Define the matrices $W_{k,l_{k}}=w_{k,l_{k}}w_{k,l_{k}}^{H}$, $H_{k}=h_{k}^{H}h_{k}$, $W_{k,l_{k}}^{B}=w_{k,l_{k}}^{B}{\left(w_{k,l_{k}}^{B}\right)}^{H}$, $H_{k}^{B}={\left(h_{k}^{B}\right)}^{H}h_{k}^{B}$. Then, the slack variables $\gamma_{k,l_{k}}$ and $\gamma_{k,l_{k}}^{B}$ are introduced, satisfying ${\mathrm{SINR}}_{k,l_{k}}\geq \gamma_{k,l_{k}}$ and ${\mathrm{SINR}}_{k,l_{k}}^{B}\geq \gamma_{k,l_{k}}^{B}$. The rank-one constraints of $W_{k,l_{k}}$ and $W_{k,l_{k}}^{B}$ are ignored due to their non-convexity [23]. As a result, the built optimization problem was transformed into

(9a) $$\begin{array}{cc} \; & \max_{\left\{W_{k,l_{k}},W_{k,l_{k}}^{B},\gamma_{k,l_{k}},\gamma_{k,l_{k}}^{B}\right\}}R^{\prime } \end{array}$$

(9b) $$\begin{array}{cc} \; & s.t.\;\;\frac{\mathrm{Tr}(H_{k}W_{k,l_{k}})}{\sum_{k^{\prime }=1,k^{\prime }\neq k}^{K}\sum_{l_{k^{\prime }}=1}^{L_{k^{\prime }}}\mathrm{Tr}(H_{k}W_{k^{\prime },l_{k^{\prime }}})+\sum_{l=1,l\neq l_{k}}^{L_{k}}\mathrm{Tr}(H_{k}W_{k,l})+\sigma_{k}^{2}}\geq \gamma_{k,l_{k}}, \end{array}$$

(9c) $$\begin{array}{cc} \; & \;\;\;\;\;\;\;\frac{\mathrm{Tr}(H_{k}^{B}W_{k,l_{k}}^{B})}{\sum_{k^{\prime }=1,k^{\prime }\neq k}^{K}\sum_{l_{k^{\prime }}=1}^{L_{k^{\prime }}}\mathrm{Tr}(H_{k}^{B}W_{k^{\prime },l_{k^{\prime }}}^{B})+\sum_{l=1,l\neq l_{k}}^{L_{k}}\mathrm{Tr}(H_{k}^{B}W_{k,l}^{B})+{\left(\sigma_{k}^{B}\right)}^{2}}\geq \gamma_{k,l_{k}}^{B}, \end{array}$$

(9d) $$\begin{array}{cc} \; & \;\;\;\;\;\;\sum_{k=1}^{K}\sum_{l_{k}=1}^{L_{k}}\mathrm{Tr}\left(W_{{}_{k,l_{k}}}^{i}\right)\leq P_{\max},\;\forall i=1,2,\ldots ,M, \end{array}$$

(9e) $$\begin{array}{cc} \; & \;\;\;\;\;\;\sum_{k=1}^{K}\sum_{l_{k}=1}^{L_{k}}\mathrm{Tr}\left(W_{k,l_{k}}^{B}\right)\leq P_{\max}^{B}, \end{array}$$

(9f) $$\begin{array}{cc} \; & \;\;\;\;\;\;\gamma_{k,l_{k}}\geq \mu_{k,l_{k}}, \end{array}$$

(9g) $$\begin{array}{cc} \; & \;\;\;\;\;\;\gamma_{k,l_{k}}^{B}\geq \mu_{k,l_{k}}^{B}. \end{array}$$

In (9a), $R^{\prime }$ is defined as

$$\begin{array}{c} R^{\prime }=\sum_{k=1}^{K}\sum_{l_{k}=1}^{L_{k}}[z_{f\left(k\right),l_{k}}^{*}\log_{2}\left(1+\gamma_{k,l_{k}}\right)+\left(1-z_{f\left(k\right),l_{k}}^{*}\right)\log_{2}\left(1+\gamma_{k,l_{k}}^{B}\right)]. \end{array}$$

To decide the convexity of (9b) and (9c), this work showed a standard form of the convex constraint. For a convex function f(x) and a concave function h(x), the constraint in the form of f(x)<h(x) is convex. The function g(x,y)=xy is quasi concave regarding to variables *x* and *y* [24]. From the above, it is concluded that constraints (9b) and (9c) are non-convex. With some basic treatment, constraints (9b) and (9c) can be written as

(10) $$\begin{array}{cc} \; & \gamma_{k,l_{k}}\left[\sum_{k^{\prime }=1,k^{\prime }\neq k}^{K}\sum_{l_{k^{\prime }}=1}^{L_{k^{\prime }}}\mathrm{Tr}(H_{k}W_{k^{\prime },l_{k^{\prime }}})+\sum_{l=1,l\neq l_{k}}^{L_{k}}\mathrm{Tr}(H_{k}W_{k,l})+\sigma_{k}^{2}\right]\leq \mathrm{Tr}\left(H_{k}W_{k,l_{k}}\right), \end{array}$$

(11) $$\begin{array}{cc} \; & \gamma_{k,l_{k}}^{B}\left[\sum_{k^{\prime }=1,k^{\prime }\neq k}^{K}\sum_{l_{k^{\prime }}=1}^{L_{k^{\prime }}}\mathrm{Tr}(H_{k}^{B}W_{k^{\prime },l_{k^{\prime }}}^{B})+\sum_{l=1,l\neq l_{k}}^{L_{k}}\mathrm{Tr}(H_{k}^{B}W_{k,l}^{B})+(\sigma_{k}^{B}{)}^{2}\right]\leq \mathrm{Tr}\left(H_{k}^{B}W_{k,l_{k}}^{B}\right). \end{array}$$

Then, AGM inequality was used to approximate the left hand of (10) and (11), satisfying

(12) $$\begin{array}{cc} \; & \frac{\xi_{k,l_{k}}^{{}^{\prime }}}{2}{\left[\sum_{k^{\prime }=1,k^{\prime }\neq k}^{K}\sum_{l_{k^{\prime }}=1}^{L_{k^{\prime }}}\mathrm{Tr}(H_{k}W_{k^{\prime },l_{k^{\prime }}})+\sum_{l=1,l\neq l_{k}}^{L_{k}}\mathrm{Tr}(H_{k}W_{k,l})+\sigma_{k}^{2}\right]}^{2}+\frac{1}{2\xi_{k,l_{k}}^{{}^{\prime }}}\gamma_{k,l_{k}}^{2}\leq \mathrm{Tr}(H_{k}W_{k,l_{k}}), \end{array}$$

(13) $$\begin{array}{cc} \; & \frac{\xi_{k,l_{k}}^{B^{{}^{\prime }}}}{2}{\left[\sum_{k^{\prime }=1,k^{\prime }\neq k}^{K}\sum_{l_{k^{\prime }}=1}^{L_{k^{\prime }}}\mathrm{Tr}(H_{k}^{B}W_{k^{\prime },l_{k^{\prime }}}^{B})+\sum_{l=1,l\neq l_{k}}^{L_{k}}\mathrm{Tr}(H_{k}^{B}W_{k,l}^{B})+(\sigma_{k}^{B}{)}^{2}\right]}^{2}+\frac{1}{2\xi_{k,l_{k}}^{B^{{}^{\prime }}}}{\left(\gamma_{k,l_{k}}^{B}\right)}^{2}\leq \mathrm{Tr}(H_{k}^{B}W_{k,l_{k}}^{B}), \end{array}$$

where $\xi_{k,l_{k}}^{{}^{\prime }}$ and $\xi_{k,l_{k}}^{B^{{}^{\prime }}}$ are updated by the following rules

(14) $$\begin{array}{cc} \; & \xi_{k,l_{k}}^{{}^{\prime }}=\frac{\gamma_{k,l_{k}}^{\left(n\right)}}{\sum_{k^{\prime }=1,k^{\prime }\neq k}^{K}\sum_{l_{k^{\prime }}=1}^{L_{k^{\prime }}}\mathrm{Tr}(H_{k^{\prime }}W_{k^{\prime },l_{k^{\prime }}}^{\left(n\right)})+\sum_{l=1,l\neq l_{k}}^{L_{k}}\mathrm{Tr}(H_{k}W_{k,l}^{\left(n\right)})+\sigma_{k}^{2}}, \end{array}$$

(15) $$\begin{array}{cc} \; & \xi_{k,l_{k}}^{B^{\prime }}=\frac{{\left(\gamma_{k,l}^{B}\right)}^{\left(n\right)}}{\sum_{k^{\prime }=1,k^{\prime }\neq k}^{K}\sum_{l_{k^{\prime }}=1}^{L_{k^{\prime }}}\mathrm{Tr}\left[H_{k}^{B}{\left(W_{k^{\prime },l_{k^{\prime }}}^{B}\right)}^{\left(n\right)}\right]+\sum_{l=1,l\neq l_{k}}^{L_{k}}\mathrm{Tr}\left[H_{k}^{B}{\left(W_{k,l}^{B}\right)}^{\left(n\right)}\right]+(\sigma_{k}^{B}{)}^{2}}. \end{array}$$

In (14) and (15), $\gamma_{k,l_{k}}^{\left(n\right)}$, ${\left(\gamma_{k,l_{k}}^{B}\right)}^{\left(n\right)}$, $W_{k,l_{k}}^{\left(n\right)}$ and ${\left(W_{k,l_{k}}^{B}\right)}^{\left(n\right)}$ indicate the optimal values of $\gamma_{k,l_{k}}$, $\gamma_{k,l_{k}}^{B}$, $W_{k,l_{k}}$ and $W_{k,l_{k}}^{B}$ after the *n*-th iteration, respectively. With all the above transformations, the optimization problem finally becomes

(16a) $$\begin{array}{cc} \; & \max_{\left\{W_{k,l_{k}},W_{k,l_{k}}^{B},\gamma_{k,l_{k}},\gamma_{k,l_{k}}^{B}\right\}}R^{\prime }\\ s.t. & \;\;\left|\right|[\sqrt{2\xi_{k,l_{k}}^{{}^{\prime }}}\left[\sum_{k^{\prime }=1,k^{\prime }\neq k}^{K}\sum_{l_{k}=1}^{L_{k}}\mathrm{Tr}(H_{k}W_{k^{\prime },l_{k^{\prime }}})+\sum_{l=1,l\neq l_{k}}^{L_{k}}\mathrm{Tr}(H_{k}W_{k,l})+\sigma_{k}^{2}\right], \end{array}$$

(16b) $$\begin{array}{cc} \; & \;\sqrt{\frac{2}{\xi_{k,l_{k}}^{{}^{\prime }}}}\gamma_{k,l_{k}},(\mathrm{Tr}\left(H_{k}W_{k,l_{k}}\right)-1)]|{|}_{2}\leq \mathrm{Tr}\left(H_{k}W_{k,l_{k}}\right)+1,\\ \; & \;\;\left|\right|[\sqrt{2\xi_{k,l_{k}}^{B^{{}^{\prime }}}}\left[\sum_{k^{\prime }=1,k^{\prime }\neq k}^{K}\sum_{l_{k^{\prime }}=1}^{L_{k^{\prime }}}\mathrm{Tr}(H_{k}^{B}W_{k^{\prime },l_{k^{\prime }}}^{B})+\sum_{l=1,l\neq l_{k}}^{L_{k}}\mathrm{Tr}(H_{k}^{B}W_{k,l}^{B})+(\sigma_{k}^{B}{)}^{2}\right], \end{array}$$

(16c) $$\begin{array}{cc} \; & \;\sqrt{\frac{2}{\xi_{k,l_{k}}^{B^{{}^{\prime }}}}}\gamma_{k,l_{k}}^{B},(\mathrm{Tr}\left(H_{k}^{B}W_{k,l_{k}}^{B}\right)-1)]|{|}_{2}\leq \mathrm{Tr}\left(H_{k}^{B}W_{k,l_{k}}^{B}\right)+1, \end{array}$$

(16d) $$\begin{array}{cc} \; & \;\;\sum_{k=1}^{K}\sum_{l_{k}=1}^{L_{k}}\mathrm{Tr}(W_{k,l_{k}}^{i})\leq P_{\max},\forall i=1,2,\ldots ,M, \end{array}$$

(16e) $$\begin{array}{cc} \; & \;\;\sum_{k=1}^{K}\sum_{l_{k}=1}^{L_{k}}\mathrm{Tr}(W_{k,l_{k}}^{B})\leq P_{\max}^{B}, \end{array}$$

(16f) $$\begin{array}{cc} \; & \;\;\gamma_{k,l_{k}}\geq \mu_{k,l_{k}}, \end{array}$$

(16g) $$\begin{array}{cc} \; & \;\;\gamma_{k,l_{k}}^{B}\geq \mu_{k,l_{k}}^{B}, \end{array}$$

where (16b) and (16c) are the second-order cone forms of inequalities (12) and (13), respectively. To this end, the original problem was transformed into the second-order cone programming (SOCP) problem, which is convex and can be readily solved by CVX solvers.

The detailed steps for solving problem (16) are summarized in Algorithm 1. Firstly, we can find the feasible solutions of $W_{k,l_{k}}$ and $W_{k,l_{k}}^{B}$, and calculate the values $\xi_{k,l_{k}}^{{}^{\prime }}$ and $\xi_{k,l_{k}}^{B^{{}^{\prime }}}$ by (14) and (15) easily. Detailed steps can refer to Algorithm 1. After the optimal values of $W_{k,l_{k}}$ and $W_{k,l_{k}}^{B}$ are attained, if the ranks of the beamforming matrices are 1, the optimal beamforming vectors can be generated by applying the eigenvalue decomposition. Otherwise, the Gaussian randomization [23] is helpful for gaining the corresponding beamforming vectors.

The analysis of the computational complexity of the transformed problem (16) can refer to reference [25], where the SOCP problem was formulated and solved. By following similar methods, the required number of iterations and the complexity for each iteration can be obtained, and then the overall computational complexity is attained.
**Algorithm 1** The proposed algorithm for solving problem (16)(1) Initialization: Set **n=1,** and the maximum number of iteration is *N*. Define the threshold δ≪Δ, where Δ is large enough.(2) Find feasible ($W_{k,l_{k}}^{\left(1\right)}$, $\gamma_{k,l_{k}}^{\left(1\right)}$, ${\left(W_{k,l_{k}}^{B}\right)}^{\left(1\right)}$, ${\left(\gamma_{k,l_{k}}^{B}\right)}^{\left(1\right)}$) to (16).(3) Calculate $\xi_{k,l_{k}}^{{}^{\prime }}$ and $\xi_{k,l_{k}}^{B^{{}^{\prime }}}$ by (14) and (15).(4) While (n⩽NandΔ>δ) (a) Solve the problem (18) with $\xi^{{}^{\prime }}$ and $\xi_{B}^{{}^{\prime }}$, and obtain the optimal solutions $W_{k,l_{k}}^{*},\gamma_{k,l_{k}}^{*},{\left(W_{k,l_{k}}^{B}\right)}^{*}$ and ${\left(\gamma_{k,l_{k}}^{B}\right)}^{*}$; (b) Calculate the optimal sum rate R(n); (c) Update $\xi_{k,l_{k}}^{{}^{\prime }}$ and $\xi_{k,l_{k}}^{B^{{}^{\prime }}}$ by (14) and (15); (d) Δ=|R(n)−R(n−1)|, and n=n+1.(5) end(6) Set $R^{*}=R\left(n-1\right)$.(7) If rank($W_{k,l_{k}}^{*}$) = 1 or rank(${\left(W_{k,l_{k}}^{B}\right)}^{*}$) = 1, use eigenvalue decomposition to obtain the optimal beamforming vectors. Otherwise, apply Gaussion randomization to obtain the beamforming vectors.

## 6. Simulation Results

This section shows the simulation results of the proposed algorithms for content placement and delivery. There was a set of 10 files of the same size, each of which had a size of 50 Mbits and two layers. The transmit powers of the MBS and the helper were 33 dBm and 23 dBm, respectively. The numbers of users and helpers were six and two. There were four and two antennas equipped in the MBS and each helper. To verify the superiority of the proposed schemes, the scheme without helper caching under the condition that the minimum QoS requirements of users is satisfied was adopted as the benchmark scheme. All results were averaged over 100 channel realizations.

### 6.1. Simulation Results of Content Placement

**Figure 3:**
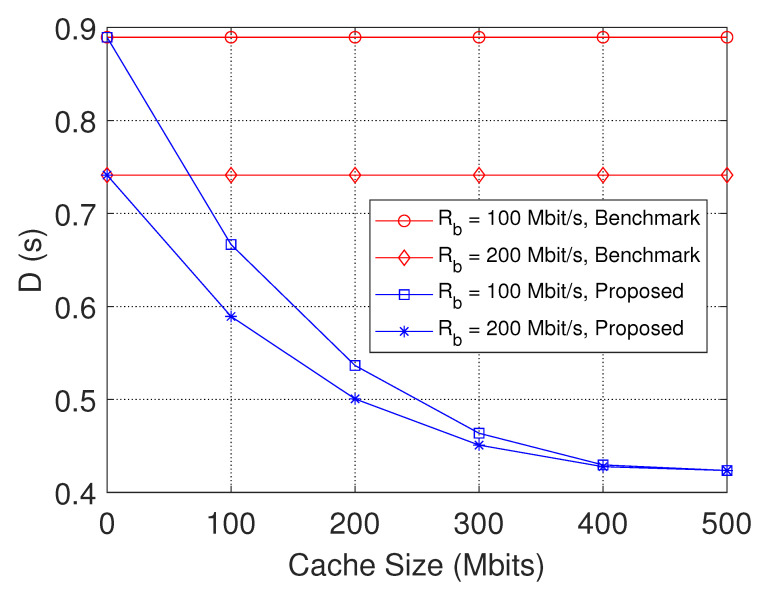
The delay performance with varying cache sizes of helpers.

From Figure 3, we can see that the minimum maximal transmission latency decreases as the cache size grows. This is because the larger the cache size, the more requested files can be cached in the helpers. More files requests can be satisfied by the local caches of helpers, and thereby the requested ones are less likely to be obtained from the MBS. The delay caused by content retrieval from the core network through the backhaul link is largely reduced. We can also find that the transmission latency decreases as the backhaul link rate increases.

**Figure 4:**
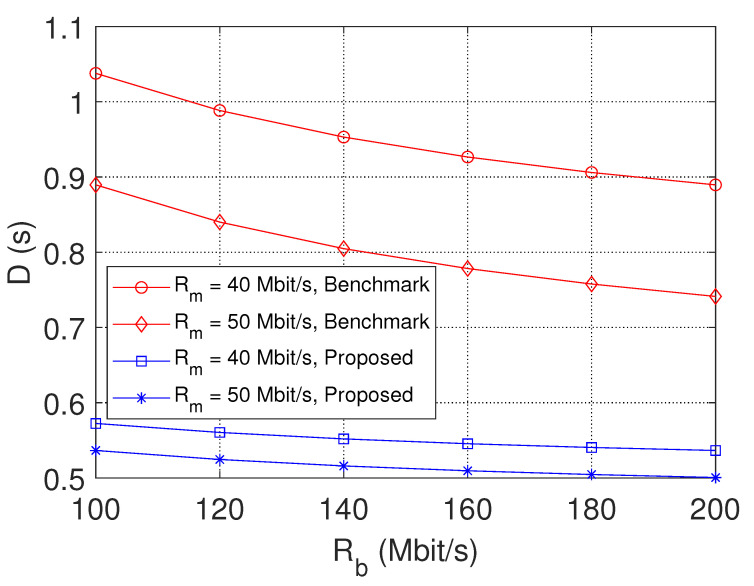
The delay performance with varying $R_{b}$.

Figure 4 shows the performance of transmission latency with varying $R_{b}$. The transmission latency will be reduced to a lower level when the transmission rate is increased. Similarly, as the transmission rate increases, the transmission delay decreases accordingly. This is because, with the increase of $R_{b}$, the backhaul capacity has a less negative effect on the content transmission. With an increased backhaul rate, the total delay is also reduced. The increasing backhaul rate can meet the increasing capacity and higher data rate requirements. When $R_{b}$ reaches a certain level, backhaul capacity will no longer be a limiting factor for large-scale video distributions.

### 6.2. Simulation Results of Content Transmission

**Figure 5:**
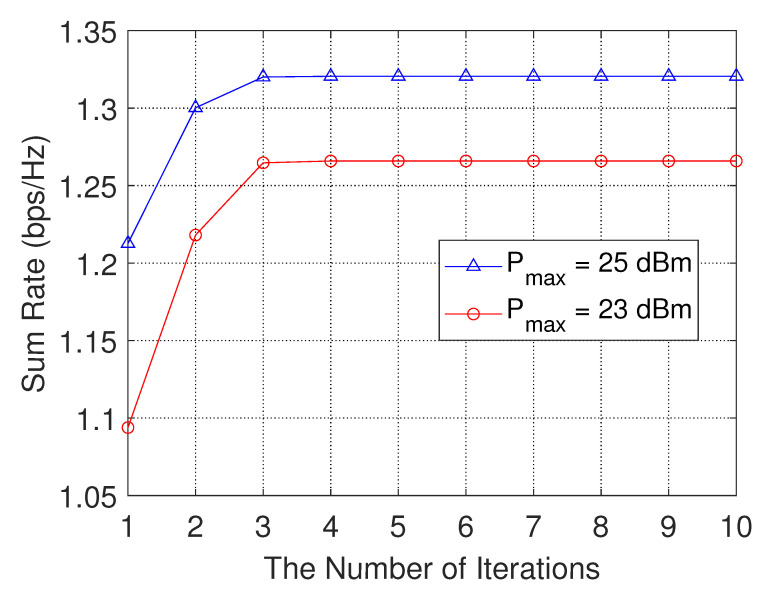
Convergence behavior of Algorithm 1.

We show the convergence property of problem (16) in Figure 4. It can be seen from Figure 5 that the proposed algorithm can converge quickly after a small number of iterations. With a smaller transmit power of the helper, the sum rate will be lower. When we further increase the power of the helper $P_{\max}$, the sum rate will be maintained at a stable value. As the transmit power of the helper increases, though the interference from helpers increases, the received signal strength from the serving helpers increases, and thus the user’s transmission delay decreases.

**Figure 6:**
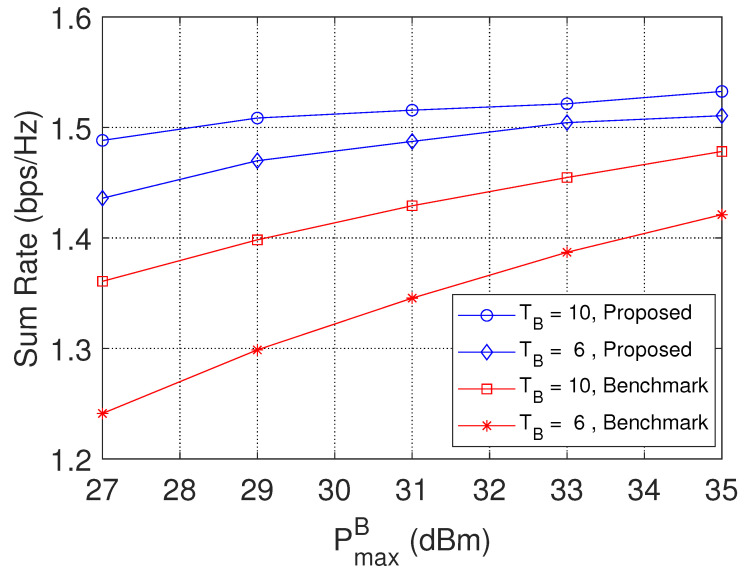
The sum rate performance with varying $P_{\max}^{B}$.

Figure 6 presents the sum rate performance with the varying transmit power of the MBS. With the increase of $P_{\max}^{B}$, there will be more power for data transmission, thus increasing the sum rate. For two different numbers of antennas equipped in the MBS, we can observe that more antennas will yield a higher sum rate. This is because more antennas can lead to a greater degree of freedom for the user, and thus the sum rate will be increased. It was also found that the sum rate performance of the proposed scheme is superior to the benchmark scheme. This is because wireless caching can bring contents closer to users and improve the sum rate performance.

## 7. Conclusions

In this paper, we investigated the optimal designs of cache placement and transmission in a heterogeneous network with caching helpers. In the cache placement phase, we formulated and solved the minimization problem of maximal transmission delay caused by each SVC layer. We also optimized the sum rate to obtain the transmit beamforming vectors of the MBS and helpers in the content transmission phase. The non-convex sum rate problem was solved by applying SDR, SCA, and AGM inequality. Simulation results showed the convergence of the proposed algorithm for solving the sum rate problem and proved the superiority of the proposed helper-assisted caching scheme. In our future work, we will consider imperfect channel state information, making channel conditions more realistic. Additionally, in the content transmission stage, we intend to incorporate NOMA to improve the spectral efficiency. 

## Data Availability

Not applicable.

## Abbreviations

The following abbreviations are used in this manuscript:

QoS	Quality of service
SVC	Scalable video coding
MBS	Macro-cell base station
SDR	Semi-definite relaxation
SCA	Successive convex approximation
AGM	Arithmetic-geometric mean
6G	Sixth generation
CRANs	Cloud radio access networks
NOMA	Non-orthogonal multiple access
SDV	Standard definition vedio
HDV	High definition vedio
BL	Base layer
EL	Enhancement layer
D2D	Device-to-device
M-Zipf	Mandelbrot-Zipf
AGWN	Additive Gaussian white noise
SINR	Signal-to-interference-plus-noise ratio
SOCP	Second-order cone programming
SAGIN	Space-air-ground integrated networks
QoE	Quality of experience
